# Stress and sleep quality in medical students: a cross-sectional study from Vietnam

**DOI:** 10.3389/fpsyt.2023.1297605

**Published:** 2023-11-13

**Authors:** Duc-Si Tran, Duy-Thai Nguyen, Thai-Hang Nguyen, Cao-Thinh-Phuoc Tran, Sy Duong-Quy, Thanh-Hiep Nguyen

**Affiliations:** ^1^Sleep Lab Unit, University of Medicine Pham Ngoc Thach, Ho Chi Minh City, Vietnam; ^2^National Institute for Control of Vaccines and Biologicals, Hanoi, Vietnam; ^3^Department of Physiology, Vietnam University of Traditional Medicine, Hanoi, Vietnam; ^4^BioMedical Research and Sleep Lab Center, Lam Dong Medical College, Dalat, Vietnam

**Keywords:** COVID-19, medical students, stress, sleep quality, Vietnam

## Abstract

**Background:**

The COVID-19 pandemic has resulted in significant global social and economic disruptions, as well as changes in personal attitude and behavior. The purpose of this research is to assess the sleep quality and stress levels of medical students.

**Method:**

Data was collected from medical students over the course of a month in 2021. A total of 4,677 students at the University of Medicine Pham Ngoc Thach were invited to complete an anonymous web-based survey, which included the Pittsburgh Sleep Quality Questionnaire Index (PSQI) for measuring sleep quality and the COVID-19 Student Stress Questionnaire (CSSQ) for evaluating stress.

**Results:**

A total of 1,502 students participated in our survey. More than half of the participants exhibited poor quality of sleep as indicated by their PSQI score. Many students reported going to bed after midnight and spending time on their smartphones. Among the students surveyed, 21.84% experienced low levels of stress (CSSQ ≤6), 63.38% had mild stress (7 ≤ CSSQ score ≤ 14), 14.78% reported high levels of stress (CSSQ >14).

**Conclusion:**

This study showed a high prevalence of poor sleep quality in the surveyed students, which could be attributed to changes in their behavior following the COVID-19 outbreak. Mild stress was also frequently observed, and it may be related to sleep disorders in this population. These important findings provide valuable insights for making recommendations, including lifestyle modifications to improve sleep quality.

## 1. Introduction

COVID-19, or Coronavirus disease 2019, has had significant impacts on not only businesses and the economy but also on people’s health and daily lives. Social distancing and home quarantine measures have been recommended by the World Health Organization (WHO) to slow the spread of the virus. Unfortunately, these measures have resulted in the suspension of outdoor physical activities, leading to an increase in sedentary behavior, screen addiction, and disrupted sleep patterns. All of which have adverse effects on global public health (1–4). A study in Spain by Borja Sañudo et al. highlighted how the COVID-19 lockdowns changed individuals’ routines, limited physical activities and increased time spent at home (5). Also, the global implementation of social distancing measures and infection control regulations have had drastic impacts on medical education, with medical institutions forced to adapt and, in some cases, transition to online teaching entirely (6).

Other studies in Europe revealed that moderate to vigorous physical activity decreased during home confinement. In Spain, research has shown sedentary behavior was linked to smartphone use patterns. The screen usage can alleviate social isolation, it also negatively influences the levels of physical activity, sedentary behavior, and sleep patterns. In fact, sleep disturbance affects 11 to 26% of the population, depending on demographics (7).

The COVID-19 pandemic has brought forth a profound connection between sleep and mental health, with both exerting significant influence on one another. Mental health issues have surged across various demographics during this challenging period, with anxiety disorders and depression affecting 30–40% of individuals affected by COVID-19. Research conducted by Groff et al. revealed that approximately one-third of COVID-19 patients received treatment for generalized anxiety disorder, one-fourth for sleep disorders, one-fifth for depression, and one in eight cases for post-traumatic stress disorder in 2021 (8). A study of 62,354 COVID-19 patients in the United States estimated that 18.1% developed psychiatric disorders within 14 to 90 days of follow-up (9). Medical students, burdened by their intensive academic workload and responsibility to support strained medical services during the pandemic, have faced heightened stress levels and compromised sleep quality. A web-based cross-sectional survey conducted in Bangladesh unveiled that over two-thirds of students reported experiencing mild to severe depression (82.4%) and anxiety (87.7%) (10).

Vietnam, a neighbor country of China, has been one of the most affected countries worldwide. According to the Coronavirus Disease Dashboard of Vietnamese Ministry of Health by the end of 2022 more than twenty thousand COVID-19 related deaths was recorded in Ho Chi Minh City despite effective epidemic prevention and control measures, including varying degrees of confinement and social distancing since 2020. These measures have disrupted both social and economic spheres, as well as induced notable changes in personal attitudes and behavior. Therefore, we seek to assess the consequences, particularly stress and sleep disorders, among medical students at Pham Ngoc Thach Medical University, Vietnam.

## 2. Method

We conducted a descriptive cross-sectional study using a self-reported questionnaire, with a target population of 4,677 medical students. An internal email containing a questionnaire link, a consent form, and an explanation of the study’s objectives and participation instructions was sent to all students. Each student, using their internal email account, was allowed to complete the questionnaire once. Participation in the survey was voluntary, and students had the freedom to decline or exit the survey at any point. No personal identifying information, such as names or email addresses, was collected in the survey.

Data collection took place over April 1st to May 1st, 2021, when the COVID-19 lockdown was partially introduced. During this period, the students were still engaged in partial online learning through their personal internal accounts and email, with limited access to social activities.

### 2.1. PSQI measures

To assess sleep quality, we employed the Pittsburgh Sleep Quality Index (PSQI), a validated tool for screening sleep disorders that was translated and tested in Vietnam in 2014.

The PSQI consists of 7 components, each scored on a scale from 0 to 3 points. A score of 0 represents no sleeping difficulty, while a higher score is related to increased sleep problems. The 7 components are cited as follows: (1) subjective sleep quality (very good vs. very poor); (2) sleep latency (≤15 min to >60 min); (3) sleep duration (≥7 h to <5 h); (4) sleep effective (≥85 to <65% h sleep/h in bed); (5) sleep disturbances (not during the past month to ≥3 times per week); (6) use of sleeping medications (none to ≥3 times a week) and (7) daytime dysfunction (not a problem to a very big problem).” All 7 items were combined to create the total PSQI score, which ranges from 0 to 21 points. As proposed by Buysse et al. (11), we categorized the results into two groups: good quality of sleep (total PSQI score ≤ 5) and poor quality of sleep (total PSQI score > 5).

### 2.2. COVID-19 stress scales

The COVID-19 Student Stress Questionnaire (CSSQ) was specifically developed to assess university students’ perceived stress during the COVID-19 pandemic lockdown. It consists of 7 items measured on a 5-point Likert scale, ranging from zero (“Not at all stressful”) to four (“Extremely stressful”). Each item was designed to cover different domains that may have been affected by the COVID-19 pandemic lockdown, and, therefore, could potentially be sources of stress. These domains include the risk of contagion, social isolation, relationship with relatives, relationship with colleagues, relationship with professors, academic studying, couple’s relationship, intimacy and sexual life. The scale provides a Global Stress score ranging from 0 to 28 (12).

Additionally, students were asked to report their levels of physical activity, sedentary behavior, and smartphone usage. They were requested to identify the top three daily activities that consumed most of their time, as well as their three most preferred activities on smartphones.

### 2.3. Data analysis

Survey responses from Google form were exported, then transcribed and analyzed using Stata software. Descriptive statistics, including average, standard deviation (SD), student’s T-test (t), chi-square test and Pearson correlations (r) were calculated.

## 3. Results

Among the 4,677 medical students enrolled at the University of Medicine Pham Ngoc Thach, we received responses from 1,502 participants, representing a 32.18% participation rate. Our survey included students from all years of the medicine program, ranging from the 1st to the 6th year. The average age of participating students was 19.5 years old, with a standard deviation of ±1.99 years. The gender ratio of the respondents was 0.81 males to 1 female.

### 3.1. Daily activities patterns

The top two preferred daily activities among the participants were web surfing or using other information technologies (72.5%) and engaging in learning activities (57.2%). When it came to smartphone usage, chatting (84.3%) and connecting to social networks (82.6%) were the most favored activities. Interestingly, learning medicine ranked fifth (41.3%), trailing behind watching entertainment clips (55.5%) and checking email (47.2%).

Only a small percentage of students (1.0%) reported smoking, while the majority consumed coffee in moderation. Specifically, 81.82% of students consumed less than one cup of coffee daily, 14.98% consumed one cup, 2.87% consumed two or three cups, and 0.33% consumed four cups or more. Similar consumption patterns were observed for tea, with rates of 81.69, 11.78, 4.86, and 1.67% for each respective category.

Of the 1,502 surveyed students, 15.98% (240 students) engaged in vigorous physical activity, 47.40% (712 students) in moderate, and 36.62% (550 students) in mild physical activity.

### 3.2. Stress level in students

According to the results of CSSQ, 21.84% of the students in our survey were classified as having a low risk of stress (CSSQ score ≤ 6), while 63.38% had a medium risk (7 ≤ CSSQ score ≤ 14), and 14.78% were deemed to have a high risk of stress. The average CSSQ score was 10.43 (±5.07), and component scores can be found in Table 1.

**Table 1:** Result of Student Stress Questionnaire.

The CSSQ subscales	Mean ± SD	Range
Relationships and academic life	4,76 **±** 3,54	0–16
Isolation	3,06 **±** 1,78	0–8
Fear of contagion	2,61 **±** 1,16	0–4
Total score	10,43 **±** 5,07	0–28

### Stress and related factors

Stress levels among students were found to be associated with grades (*p* < 0.001) and disciplines (*p* = 0.03). Additionally, having a chronic disease (*p* = 0.01), consuming coffee (*p* = 0.018), and consuming more than one cup of tea per day (*p* = 0.003) were also statistically linked to stress levels.

### 3.3. Sleep quality and related factors

**Figure 1:**
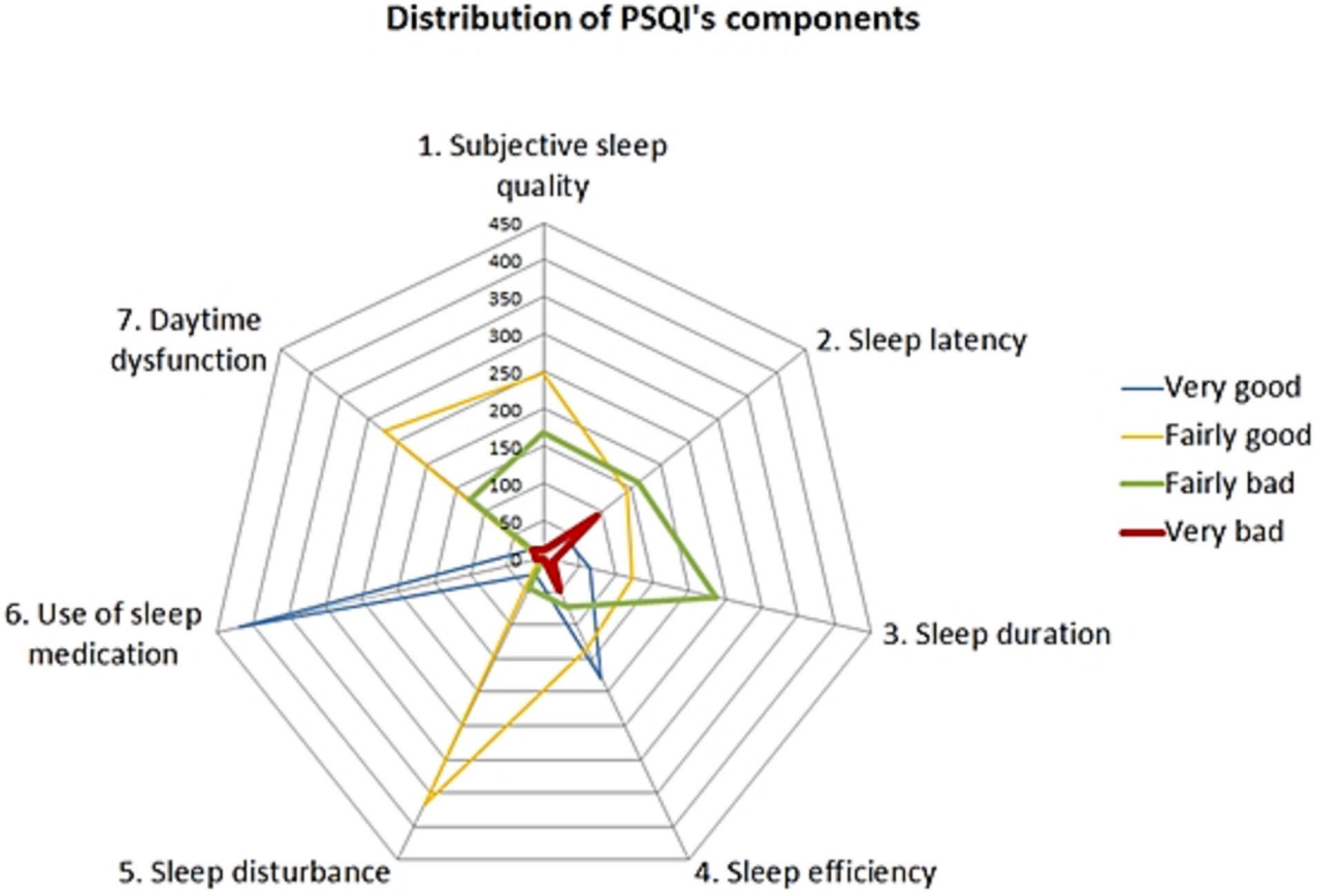
Detailed result of PSQI.

Based on the results obtained from the PSQI assessment (Figure 1), approximately 49.73% of the participants demonstrated good sleep quality (PSQI score lower than 6 points). The mean PSQI score in our study was 5.8, with a standard deviation of 2.84. All seven components of sleep quality were significantly affected (*p* ≤ 0.01), with notable changes observed in sleep latency, sleep efficiency, and sleep duration. The duration of time spent in bed varied greatly among the students, ranging from 0.5 to 15.5 h per day, with an average of 7.68 h (±SD: 1.71). The average sleep latency was 23.92 min, and the mean sleep efficiency was 89.80%. Approximately 42.61% of students reported going to bed at midnight or later. Moreover, 13.05% of students spent 9 h or more in bed per day, and 1.07% even exceeded 12 h. Students who went to bed at midnight or later exhibited higher PSQI scores, with a mean score of 6.36 (±2.94) compared to 5.39 (±2.69) for those who went to bed earlier, indicating a statistically significant difference (*p* < 0.001). The late bedtimes were associated with shorter overall time spent in bed (7.23 vs. 8.02 h, *p* < 0.001) and reduced actual sleep time (mean time: 6.52 vs. 7.05 h, *p* < 0.001). Conversely, students who went to bed later experienced longer sleep latency, indicating difficulty falling asleep (mean latency: 39.06 vs. 23.94, *p* < 0.001). Interestingly, these students demonstrated higher sleep efficiency compared to others (90.90% vs. 88.93%, *p* = 0.02).

In addition to studying sleep habits, we explored other lifestyle factors. A significant correlation was found between poor sleep quality and consuming two or more cups of coffee daily (*K*^2^ = 8.39, *p* = 0.004). Similarly, a significant relationship was observed with the consumption of one or more cups of tea daily (*K*^2^ = 8.77, *p* = 0.004). The majority of the medical students did not smoke, so the relationship between sleep quality and smoking was not examined. Furthermore, students with chronic diseases exhibited higher PSQI scores compared to those without chronic conditions (6.73 vs. 5.75, *p* = 0.002). Multiple regression analyses revealed associations between later sleep times (*p* < 0.001), tea consumption (*p* = 0.002), and sleep quality.

### Sleep quality and the relation with stress

The level of stress experienced during the COVID-19 pandemic had an adverse effect on sleep quality (*p* < 0.001). The linear regression equation for PSQI score and CSSQ score was as follows: PSQI = 4.69568 + 0.10599 * CSSQ. Except for the “Fear of contagion” component, all other six components of the CSSQ were significantly correlated with the PSQI score (*p* < 0.001). Therefore, sleep quality was found to be related to two CSSQ subscales: Relationships and academic life (*p* < 0.001) and Isolation (*p* < 0.001). By utilizing multivariable regression, the equation can be expressed as follows: PSQI score = 4.7837 + 0.1139 * (Relationships & academic life) + 0.0155 * (Isolation).

## 4. Discussion

The COVID-19 pandemic has caused widespread social and economic disruptions, leading to significant changes in personal attitudes and behaviors (4). Inadequate sleep not only impairs concentration and effective learning but also contributes to feelings of stress among students. Research has consistently shown that students who fail to obtain the recommended 8–10 h of sleep per night are more prone to experiencing stress compared to those who do.

A study was conducted at six Jordanian medical schools via an online survey to examine the effects of COVID-19 on various life activities. The findings revealed that 66% of the students expressed concerns regarding the well-being of their family members, and 58.4% highlighted their apprehensions about the disruption of clinical sessions and laboratory access. Approximately half of the participants experienced severe mental distress, with physical fitness, exercise, and studying being particularly affected during the COVID-19 pandemic (10, 13).

Notably, a modest delay of 25 min in school start time was associated with improvements in sleep duration, daytime sleepiness, mood, and caffeine use. However, when the original (earlier) school start time was reinstated, sleep duration reverted to baseline levels. These findings hold significant implications for public policy and further support the notion that aligning school schedules with adolescents’ circadian rhythms and sleep needs can yield health benefits (14).

Moreover, the COVID-19 pandemic has introduced additional challenges for students, including disruptions to routines and adaptation to new pedagogies and environments. Establishing a consistent routine that incorporates online classes, dedicated study time, and a regular sleep schedule can provide valuable guidance throughout the day. Any changes to this routine can make it more challenging for students to manage their time effectively, leading to increased stress levels. In our study, a significant portion of participants consists of first-year students. The transition to a new pedagogical approach, such as online learning, or the shift from high school to university can be an inherently stressful period. The introduction of new classes, teachers, and routines requires an adjustment period for students, adding to the potential stress they may experience.

During the COVID-19 lockdown, individual routines had to adapt to the new situation, as the model for web-based online learning education replaced traditional physical clinical teachings. In Iran, students predominantly engaged in mobile and computer games (30.1%), followed by studying (26.6%), and watching television (13.8%) (2). In our study, the top three daily preferences among students during the same period were medical learning (79.15%), web surfing (65.0%), and watching television or video clips (37.1%). This result showed medical learning was the primary preference for the surveyed students but they had to utilize laptops or smartphones to complete their courses, which naturally facilitated the use of screen devices. Interestingly, we observed that students used smartphones more for connecting to social networks and chatting rather than for learning medicine. This finding supports our initial assumption and raises the hypothesis that smartphones may be less conducive to effective online learning.

Recent evidence indicates a significant increase in sedentary behavior during the pandemic lockdown, with varying levels of physical activity declining across countries due to different government measures. Factors such as cultural norms, population characteristics, and home environment also contributed to this trend. Our survey was conducted during a partial lifting of the lockdown, before students returned to university, resulting in low physical activity levels. Unfortunately, we lack comparative data from previous periods. Additionally, our study did not find a significant relationship between PSQI and physical activity volume. However, a 2017 study by Podhorecka et al. demonstrated a significant correlation between early falling asleep, nighttime awakenings, and engaging in intense or moderate physical activity. It also revealed that individuals’ participation in such activities experienced fewer sleep disruptions, faster sleep onset, and reported better sleep quality (15).

Sleep significantly impacts an individual’s overall health, and sleep disorders have become a growing public health concern (16, 17). Although sleep disorders are more common among older individuals, the lifestyle of young adults, with academic pressures and extensive use of electronic media, poses a significant risk, especially when combined with social distancing measures (18, 19). Among various subgroups, medical students are particularly vulnerable to poor sleep quality due to the demanding nature of their studies, long working hours, emotionally challenging tasks, and lifestyle changes (20, 21). More than half of our participants exhibited poor sleep quality according to the PSQI, with all five components of the PSQI significantly affected (*p* ≤ 0.01). The most notable changes were observed in sleep latency, sleep efficiency, and sleep duration. Similar results were found by Romero-Blanco et al. in nursing students, where the PSQI score increased by approximately 1 point (from 5.5 to 6.4) and the time spent in bed increased by 1 h (from 7.6 to 8.5) during the lockdown in Spain (22). A significant number of the surveyed students (37.0%) slept for 9 h or more, and 3.4% slept for 12 h or more, although a study in Iran reported an even higher figure of (53.5%) (23). Another study conducted in Brazil also confirmed sleep deprivation among medical students, with 38.9% of the students experiencing poor sleep quality based on the PSQI (24).

Coffee and tea consumption were significantly associated with sleep quality. Despite being deeply ingrained in Vietnamese culture, both beverages act as stimulants and can negatively impact sleep. They are known to prolong sleep latency, reduce total sleep time, worsen perceived sleep quality, increase light sleep, shorten deep sleep duration, and lead to more frequent awakenings (25). Interestingly, our study reveals that consuming tea throughout the day produces alerting effects similar to coffee, even though it contains lower caffeine levels. However, tea is less likely to disrupt sleep compared to coffee (26).

Alcohol consumption and drug abuse can also have some effect on sleep disturbances (27, 28). However, we did not include these factors in the research questionnaire to avoid making it too long. This is one of the limitations of the study. Anyway, because the research subjects are medical students, few people would have bad habits that affect their health. In fact, in our study, the number of students who smoked was very small, only 1%.

Sleep deprivation negatively impacted daytime performance, as individuals were more prone to experiencing sleepiness and reduced energy levels throughout the day. Moreover, it had a negative effect on mood, leading to increased irritability and feelings of sadness. Several studies have highlighted a clear link between sleep deprivation, screen usage, and social media access. Those who experienced sleep deprivation were at a higher risk of nocturnal disruptions, likely due to the higher prevalence of computers, cell phones, and smartphones present in their bedrooms (29, 30).

Chronic diseases can affect the patient’s sleep quality, including migraine, one of the most frequent diseases in the young. A review published in 2021 showed the relation between migraine and sleep (31). However, few of our students declared suffering from migraine. In fact, we need other studies with specific questionnaires to reveal the real prevalence of migraine and its relation with sleep disorders.

Stress in medical students during COVID-19 is associated with various risk factors, including fear of contagion, isolation, and interpersonal relationships. Studies have shown that health-related fears and social isolation contribute to the psychosocial burden experienced by medical students. The proximity of medical students to teaching hospitals, which may serve as potential sources of infection for themselves and their household members, adds to their stress levels (32, 33). Our findings emphasize the importance of reducing psychological and health-related stressors, promoting specific online learning behaviors, and providing a well-established online learning environment to mitigate the negative impacts of COVID-19 on the psychosocial well-being of medical students.

In our study, except for the “Fear of contagion” component, all other six components of CSSQ were significantly correlated with the PSQI score (*p* < 0.001). The relationship between sleep quality and stress was clear. However, as our study population is medical students with basic knowledge about biology and infectious diseases, they may not have feared contagion as much as having difficulty falling asleep. Our understanding of COVID-19 has evolved since 2020, and the period when Vietnam was most affected by COVID-19 was August 2021, a few months after we finished collecting data. Another study showed sleep disorders, stress and burnout in Vietnamese nurse students working in COVID-19 isolation camps, but these were mostly related to surcharge and work-conditions (34).

In literature, the fear of contagion among Italian students increased significantly from April 2020 (mean score 1.79) to April 2021 (mean score 2.59). This score is similar to our result but other items and the total CSSQ scores were significantly higher (35). Our students seem to scope better with isolation and changes in relationships and academic life than the Italian students. However these two stress subscores are also correlated with the sleep quality of our students.

About the related factors, a systematic literature review on stress burden among dental students showed that the first related factor of stress was grades and this is similar to our result (36). However, other related factors such as the atmosphere created by clinical professors or the amount of the assigned classwork were not studied in our research. In fact, there are differences in the context and the methodology between our work and others. Aiming to study the stress related to COVID-19, we used the specific questionnaire (CSSQ). Therefore, the comparison of our results with other studies using general stress scales would be difficult.

Prior to the COVID-19 pandemic, inadequate sleep and exercise were linked to burnout and depression among medical students. Pathological sleepiness was significantly associated with a higher prevalence of burnout, while insufficient sleep was correlated with lower professional efficacy and higher exhaustion scores. Burnout was also associated with a positive depression screening. Independent predictors of burnout included a positive depression screening, pathological sleepiness, and sleeping less than 7 h per night (37). This study showed that sleep habits, exercise, and depression were factors contributing to the risk of burnout among medical students.

## 5. Conclusion

This study indicated the concerns arising from the challenges faced by students worldwide during the COVID-19 pandemic, particularly in relation to their psychophysical health. Our findings revealed significant behavioral changes among students during the COVID-19 outbreak. They experienced notable lifestyle changes during the quarantine, including reduced physical activity, increased sedentary behavior, prolonged smartphone usage, and altered sleep patterns. Stress was also high due to COVID-19, particularly the fear of contagion. However, this fear did not significantly contribute to the relationship between stress and sleep disorders. These findings have relevance in making recommendations, including potential lifestyle modifications.

### 5.1. Limitations

This study had a few limitations worth mentioning. Firstly, we employed convenience sampling methodology, which may introduce potential volunteer bias into our results. However, as all students were required to use internal email for learning, the online questionnaire was not a disadvantage but rather a matter of convenience.

Secondly, the COVID-19 pandemic has significantly disrupted lives worldwide, creating a pervasive sense of uncertainty and anxiety. That is why we used the COVID-19 Student Stress Questionnaire (CSSQ), a specifically developed scale for this situation. In turn, comparing our results with other studies using a general stress scale proved to be challenging.

## Data availability statement

The original contributions presented in the study are included in the article/supplementary material, further inquiries can be directed to the corresponding author.

## Ethics statement

The studies involving human participants were reviewed and approved by the Ethical Committee of Psychological Research of the University of Medicine Pham Ngoc Thach. The students/participants provided their written informed consent to participate in this study.

## Author contributions

D-ST: Conceptualization, Data curation, Funding acquisition, Methodology, Resources, Validation, Visualization, Writing – original draft, Writing – review & editing. D-TN: Conceptualization, Funding acquisition, Project administration, Supervision, Validation, Visualization, Writing – original draft, Writing – review & editing. T-HN: Conceptualization, Data curation, Writing – original draft. C-T-PT: Data curation, Formal analysis, Resources, Software, Writing – original draft. SD-Q: Conceptualization, Supervision, Writing – original draft, Writing – review & editing. T-HN: Conceptualization, Methodology, Visualization, Writing – original draft.
